# The plant defensin NaD1 induces tumor cell death via a non-apoptotic, membranolytic process

**DOI:** 10.1038/cddiscovery.2016.102

**Published:** 2017-01-23

**Authors:** Amy A Baxter, Ivan KH Poon, Mark D Hulett

**Affiliations:** 1 Department of Biochemistry and Genetics, La Trobe Institute for Molecular Science, La Trobe University, Melbourne, VIC 3086, Australia

## Abstract

Cationic anti-microbial peptides (CAPs) have an important role in host innate defense against pathogens such as bacteria and fungi. Many CAPs including defensins also exhibit selective cytotoxic activity towards mammalian cells via both apoptotic and non-apoptotic processes, and are being investigated as potential anticancer agents. The anti-fungal plant defensin from ornamental tobacco, *Nicotiana alata* Defensin 1 (NaD1), was recently shown to induce necrotic-like cell death in a number of tumor cell types within 30 min of treatment, at a concentration of 10 *μ*M. NaD1-mediated cell killing within these experimental parameters has been shown to occur via binding to the plasma membrane phosphatidylinositol 4,5-bisphosphate (PIP2) in target cells to facilitate membrane destabilization and subsequent lysis. Whether NaD1 is also capable of inducing apoptosis in tumor cells has not been reported previously. In this study, treatment of MM170 (melanoma) and Jurkat T (leukemia) cells with subacute (<10 *μ*M) concentrations of NaD1 over 6–24 h was investigated to determine whether NaD1 could induce cell death via apoptosis. At subacute concentrations, NaD1 did not efficiently induce membrane permeabilization within 30 min, but markedly reduced cell viability over 24 h. In contrast to other CAPs that have been shown to induce apoptosis through caspase activation, dying cells were not sensitive to a pancaspase inhibitor nor did they display caspase activity or DNA fragmentation over the 24 h treatment time. Furthermore, over the 24 h period, cells exhibited necrotic phenotypes and succumbed to membrane permeabilization. These results indicate that the cytotoxic mechanism of NaD1 at subacute concentrations is membranolytic rather than apoptotic and is also likely to be mediated through a PIP2-targeting cell lytic pathway.

## Introduction

Cationic anti-microbial peptides (CAPs), of which defensins form a major subclass, are a diverse family of innate immunity peptides that often exhibit the ability to induce cell death in a wide variety of pathogenic microbes as well as neoplastic mammalian cells.^1–3^ Thus, there is an emerging interest in developing CAPs for therapeutic applications, including the development of novel antibiotics, anti-fungal agents and anticancer drugs.^4,5^ In relation to the potential use of CAPs in anticancer therapy, CAPs make attractive candidates because of a number of unique properties that distinguish them from traditional chemotherapy options. For example, CAPs often display high selectivity for tumor cells compared with normal cells because of the higher negative cell surface charge of many tumor cells,^6,7^ whereas their nonselectivity for rapidly dividing cells, unlike most chemotherapeutics, means they have the potential to target both active and dormant tumors.^8,9^ Furthermore, CAPs often act via plasma membrane targeting and may therefore reduce the likelihood of promoting multidrug resistance in target cells, another major hindrance to current treatments.^10,11^

Although most CAPs function by acting on the plasma membrane to induce membrane permeabilization, a selection of these have the ability to enter cells and interact with intracellular factors to induce cell death via apoptosis.^12–14^ In the context of tumor cells, factors such as concentration, target cell type and treatment duration have all been reported as determinants of whether a CAP acts via direct membrane permeabilization or has the ability to modulate intracellular signaling pathways.^13–16^ From a therapeutic perspective, determining whether a CAP is capable of inducing tumor cell death via multiple means could influence important clinical strategies, such as required dosage and duration of treatment. The solanaceous plant defensin from ornamental tobacco, *Nicotiana alata* Defensin 1 (NaD1), was previously reported to induce direct tumor cell membrane lysis and shows potential for development as a novel anticancer molecule. In this study, NaD1 has been investigated for its ability to induce apoptosis in mammalian tumor cells.

Initially, NaD1 was discovered as an anti-fungal molecule, displaying potent activity towards fungal and yeast cells.^17,18^ The ability of NaD1 to induce cell death in filamentous fungi and yeast cells involves interactions with specific cell wall components, followed by membrane lysis and cellular uptake.^19^ Furthermore, NaD1 is reported to induce ROS production and activation of the HOG stress pathway in *Candida albicans*, indicating that its cytotoxic activity is not limited to membrane disruption in the fungal setting.^18^ More recently, the effects of NaD1 on mammalian cells was investigated, revealing that NaD1 displays selective toxicity towards a range of mammalian tumor cells at low micromolar concentrations.^17^ NaD1 at a concentration of 10 *μ*M induces acute cytotoxicity, defined by a range of necrotic characteristics within 30 min of NaD1 treatment. Cells treated with 10 *μ*M NaD1 exhibit rapid plasma membrane disruption accompanied by large, non-retracting membrane blebs and cytosolic leakage.^17^ This phenotype is observed in over 50% of cells following acute treatment of 10 *μ*M NaD1. For simplicity, this concentration of NaD1 will henceforth be described as the ‘acute’ concentration. The ability of NaD1 to induce cell death under these conditions has been shown to depend on an interaction between NaD1 and the plasma membrane phospholipid, phosphatidylinositol 4,5-bisphosphate (PIP2), with which NaD1 forms a oligomeric complex capable of inducing membrane lysis.^17^ Furthermore, live cell imaging studies involving the acute treatment of mammalian tumor cells with BODIPY-labeled NaD1 have shown that NaD1 rapidly enters the cytoplasm of cells upon membrane permeabilization and appears to bind to organelles including the mitochondria and nucleus.^17^ Although membrane permeabilization occurs immediately following intracellular uptake of NaD1, the specific involvement of organelles, such as the mitochondria, in the ability of NaD1 to induce cell death has not been determined. This is particularly relevant as several CAPs have been reported to induce cell death via apoptosis by interacting with the mitochondrial membrane and/or triggering the release of cytochrome *C*.^12,14,20,21^ It is of interest to note that NaD1, in addition to binding a range of phosphatidylinositol phospholipids, including mono-, di- and tri-phosphate forms, also binds to the mitochondrial phospholipid cardiolipin *in vitro*,^17^ suggesting that, if internalized, NaD1 could potentially interact with the mitochondrial membrane.

In this study, we sought to determine whether NaD1 can induce tumor cell death via a non-membranolytic mechanism(s) at subacute concentrations (that is, <10 *μ*M) over a longer period of time. NaD1 was found to inhibit the growth of a range of mammalian tumor cells over 24 h, at subacute concentrations that only induced minimal membrane permeabilization within 30 min. Whether this growth inhibition was due to apoptosis was further investigated in two different tumor cell lines (MM170 and Jurkat). Described herein, multiple lines of evidence revealed that treatment for up to 24 h with subacute concentrations of NaD1 led to a similar phenotype as reported under acute treatment settings, suggesting that cell death under these conditions was not mediated through apoptosis, but via the previously observed PIP2-dependent mechanism.

## Results

### NaD1 is cytotoxic towards mammalian tumor cells at subacute concentrations

**Figure 1:**
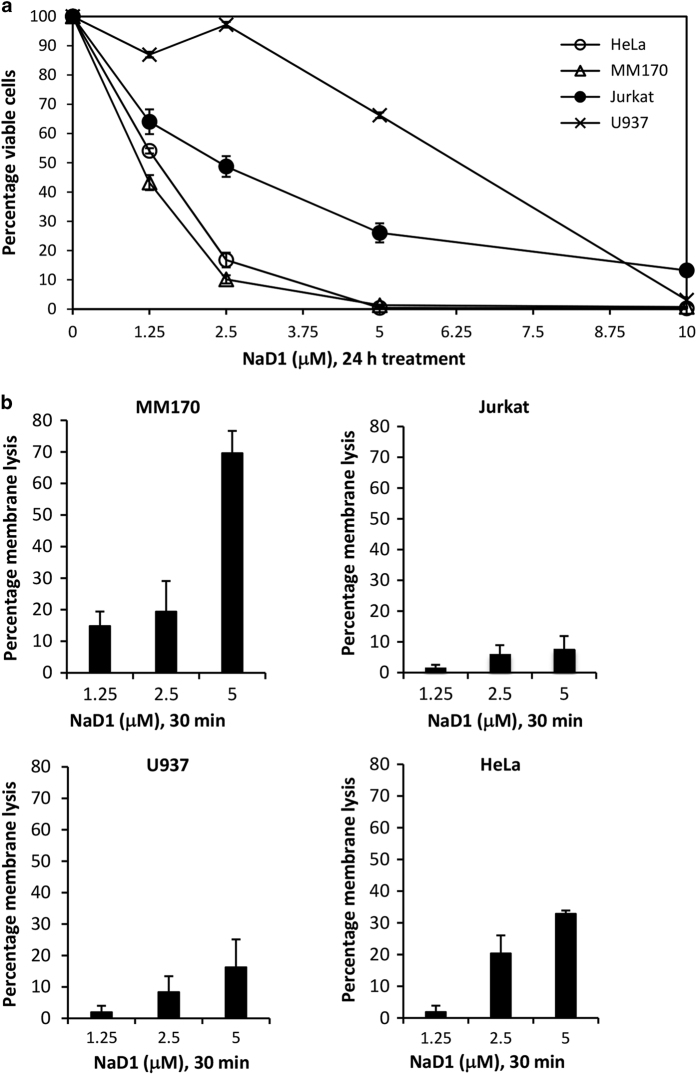
NaD1 inhibits growth of mammalian tumor cells over 24 h at subacute concentrations that do not efficiently induce rapid membrane permeabilization. The ability of NaD1 to inhibit growth of MM170, HeLa, Jurkat and U937 cells was determined using the MTT/MTS cell viability assay. Cells were treated with increasing concentrations of NaD1 (1.25, 2.5, 5 and 10 *μ*M) for 24 h, with determination of cell viability calculated as the percentage of 100% viability (untreated control samples). Half-maximal inhibitory concentration (IC_50_) values (*μ*M) were extrapolated using Excel (annotated by black dashed arrows). Data in (**a**) is representative of three independent experiments. (**b**) The release of LDH from mammalian tumor cells following 30 min treatment with NaD1 at subacute concentrations (1.25, 2.5 and 5 *μ*M) was measured via the LDH cytotoxicity assay. Data in (**b**) are representative of at least two independent experiments, error bars represent S.E.M, *n*=3.

As described previously by Poon *et al.*,^17^ NaD1 at an acute concentration (10 *μ*M) induces rapid membrane permeabilization in both adherent and non-adherent tumor cell types, accompanied by large plasma membrane blebs. This necrotic phenotype is observed within the first 30 min of NaD1 treatment.^17^ To first determine the effects of NaD1 on cell viability at subacute concentrations (<10 *μ*M) over a 24 h period, MTT (3-(4,5-dimethyl-2-thiazolyl)-2, 5-diphenyl-2*H*-tetrazolium bromide; for adherent cells) and MTS (3-(4,5-dimethylthiazol-2-yl)-5-(3-carboxymethoxyphenyl)-2-(4-sulfophenyl)-2*H*-tetrazolium, inner salt; for non-adherent cells) cell viability assays were performed on four human cell tumor lines at concentrations of 1.25, 2.5, 5 (subacute) and 10 *μ*M (acute; Figure 1a). The cell types tested were two adherent lines, cervical cancer (HeLa) and malignant melanoma (MM170) cells, and two non-adherent lines, monocytic lymphoma (U937) and leukemic T-cell (Jurkat) cells. Overall, the adherent cell lines displayed a higher degree of growth inhibition by NaD1 over 24 h compared with the non-adherent cell lines, with MM170 exhibiting the most significant growth inhibition and U937 being the least sensitive. The IC_50_ values of each cell type were 1.1 *μ*M (MM170), 1.4 *μ*M (HeLa), 2.4 *μ*M (Jurkat) and 6.3 *μ*M (U937). To determine whether the growth inhibition by NaD1 was due to rapid membrane permeabilization, the ability of subacute concentrations of NaD1 to induce membrane permeabilization within 30 min was measured by a lactate dehydrogenase (LDH) release cytotoxicity assay^17^ (Figure 1b). Interestingly, at concentrations that caused substantial reduction in cell viability over 24 h, NaD1 had minimal effects on membrane permeability over 30 min. This trend was most pronounced at the lower concentrations of 1.25 and 2.5 *μ*M across the four cell types. These data indicate that, although subacute concentrations of NaD1 can inhibit the growth of tumor cells over 24 h, these concentrations of NaD1 are not efficient at inducing direct membrane permeabilization over 30 min.

**Figure 2:**
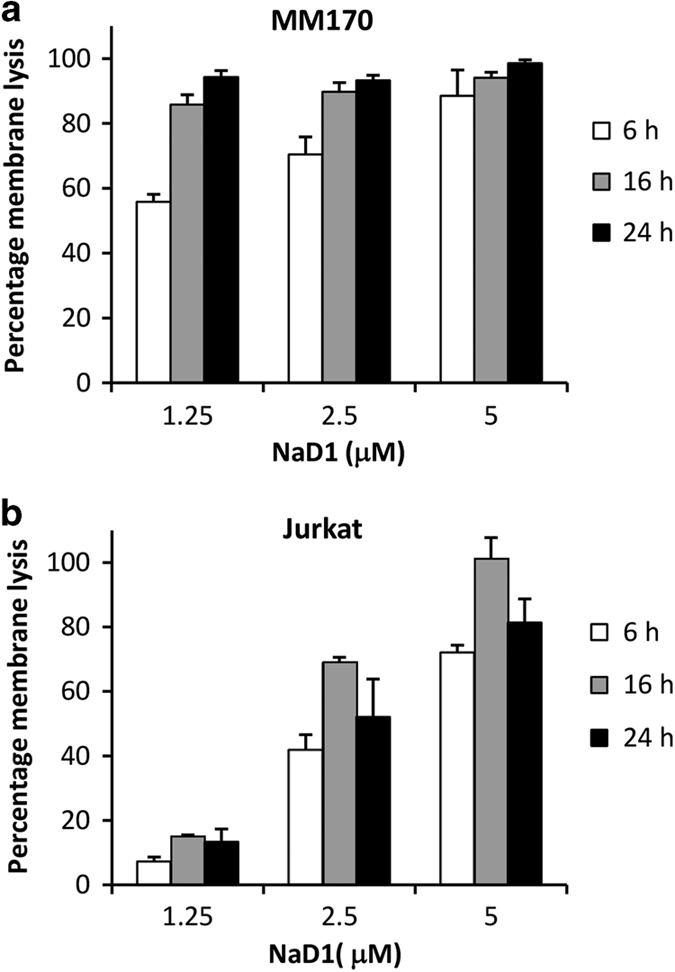
NaD1 induces membrane permeabilization of mammalian tumor cells at subacute concentrations over 24 h. The release of LDH from MM170 (**a**) and Jurkat (**b**) cells following 6, 16 and 24 h treatment with NaD1 at subacute concentrations (1.25, 2.5 and 5 *μ*M) was measured via the LDH cytotoxicity assay. Data are representative of at least two independent experiments, error bars represent S.E.M, *n*=3.

To investigate the cause of growth inhibition in tumor cells treated with subacute concentrations of NaD1, the adherent (MM170) and non-adherent (Jurkat) cell types that displayed the highest sensitivity over 24 h were selected for all subsequent studies. The ability of NaD1 to induce membrane permeabilization at subacute concentrations at 6, 16 and 24 h time points was next determined. Both MM170 and Jurkat cells showed LDH release upon exposure to NaD1 across the three time points tested, with MM170 displaying greater sensitivity towards NaD1 (Figure 2). Collectively, these data demonstrate a trend between the degree of growth inhibition and level of membrane permeabilization in both MM170 and Jurkat cells, following treatment with subacute concentrations of NaD1 over 24 h.

### NaD1-mediated cell killing at subacute concentrations is non-apoptotic

We next sought to determine whether the cytotoxic/growth inhibitory activity observed in MM170 and Jurkat cells following treatment with subacute concentrations of NaD1 could be the result of apoptosis, or whether the lower concentration of NaD1 simply delays the induction of primary necrosis.

#### NaD1 does not target the mitochondria before inducing membrane lysis

**Figure 3:**
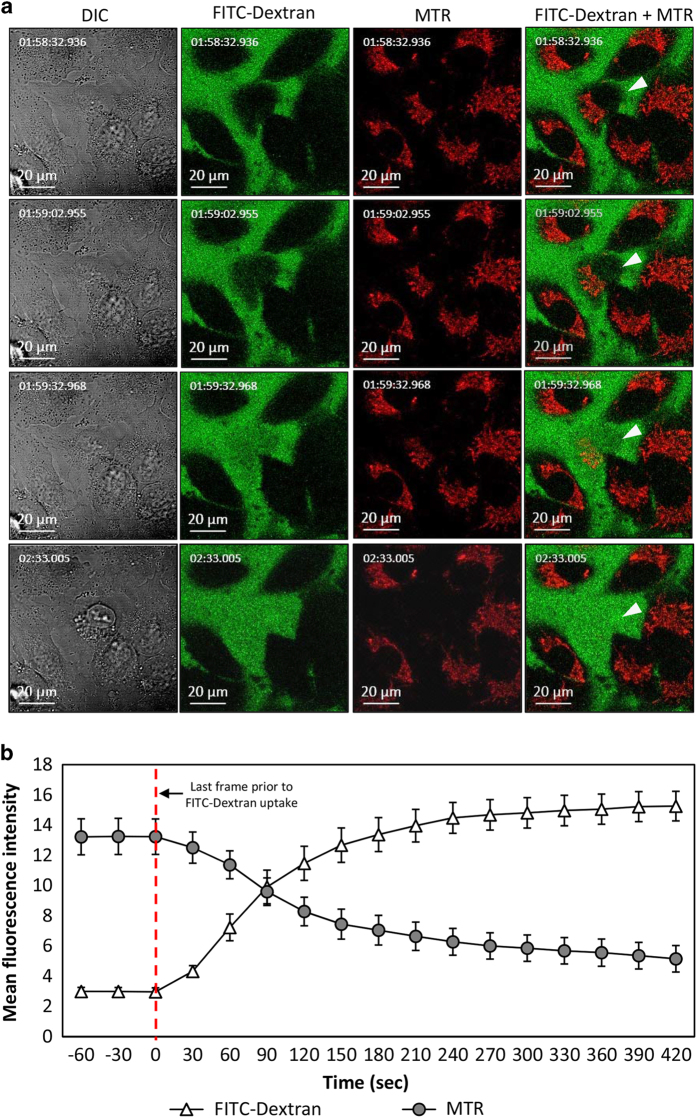
NaD1-mediated loss of mitochondrial membrane potential in MM170 cells occurs simultaneously to plasma membrane permeabilization. (**a**) The effect of NaD1 on the mitochondrial membrane potential in MM170 cells was determined by live cell imaging using confocal laser scanning microscopy (CLSM). Cells were pre-treated with MTR before being treated with 1.25 *μ*M NaD1 and imaged at 30 s intervals over 4 h in growth medium containing FITC-dextran (4 kDa). Images of key time points (taken from Supplementary Video S1) display uptake of FITC-dextran and loss of MTR fluorescence in a single cell (white arrows). Relative time is indicated in top left of each panel (h:m:s:ms). Data are representative of three independent experiments. (**b**) Kinetic analysis of mean fluorescence intensity of FITC-dextran *versus* MTR fluorescence was plotted for individual cells from 1 min before to 7 min after final baseline reading of FITC-dextran before observable uptake into the cell (Time ‘0’, indicated by red dashed line). Data in (**b**) represent average fluorescence across all cells. *n*=27, error bars represent S.E.M.

The cytotoxic activity of certain CAPs has been shown to involve mitochondrial targeting. A number of studies have reported CAP-mediated effects on the mitochondrial membrane of tumor cells within experimental parameters that are also conducive to cell death induction,^12,21^ including the loss of mitochondrial membrane potential (MMP) that specifically precedes cell death.^14,20^ To determine whether NaD1 could affect the MMP before plasma membrane lysis, MM170 were incubated with the mitochondrial stain, MitoTracker Red (MTR), before treatment with NaD1 and live cell imaging over 4 h. Although MTR does not provide a direct measure of MMP, its accumulation within the mitochondria is dependent on MMP and it can therefore be used to measure MMP indirectly.^22^ To determine the kinetics of plasma membrane permeability relative to loss in MMP (as shown by the loss of MTR fluorescence), FITC-dextran (4 kDa) was added to the extracellular media, with uptake into cells representing loss of plasma membrane integrity.^17^ In NaD1-sensitive cells, FITC-dextran could be seen entering the cytoplasm simultaneously to a reduction in MTR fluorescence (Supplementary Video S1). Selected frames from Video S1 are displayed to demonstrate changes in fluorescence in single cells undergoing membrane lysis following NaD1 treatment, indicated by white arrows (Figure 3a). Kinetic analysis of multiple individual cells at the time of cell lysis quantitatively illustrates that plasma membrane permeabilization in NaD1-sensitive cells, as determined by FITC-dextran uptake, occurred simultaneously to, rather than following, a loss in MMP in these cells (Figure 3b). These data demonstrate that MMP of NaD1-treated cells is not lost before plasma membrane lysis, suggesting that the anticancer mechanism of NaD1 is unlikely to act through a mitochondria-dependent pathway in these cell types.

#### NaD1-mediated cytotoxicity is not caspase-dependent

**Figure 4:**
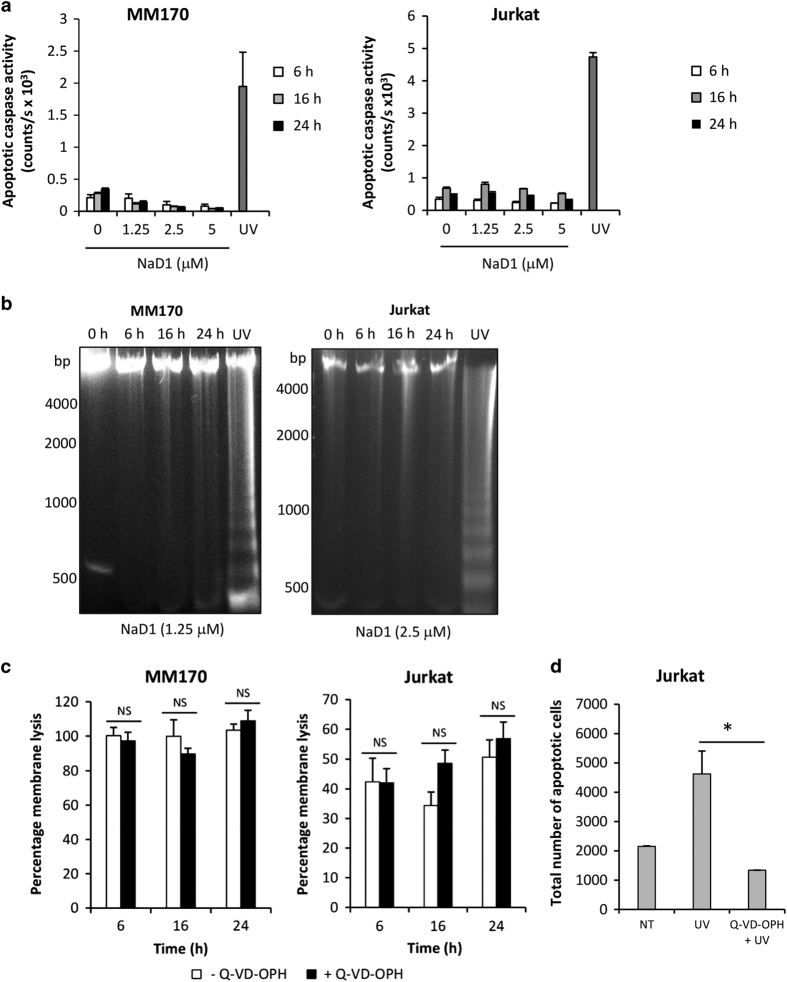
NaD1-mediated tumor cell killing at subacute concentrations is not caspase-dependent. (**a**) The ability of NaD1 to induce caspase-dependent apoptosis in MM170 and Jurkat cells was determined using the Caspase-Glo 3/7 Assay. Caspase activity expressed as bioluminescence was detected at 6, 16 and 24 h following treatment with NaD1 at 1.25, 2.5 and 5 *μ*M. NaD1 exposed to ultraviolet (UV) radiation for 4 h was used as a control. (**b**) Fragmentation of chromosomal DNA over 6, 16 and 24 h was determined by agarose gel electrophoresis following 1.25 *μ*M (MM170) or 2.5 *μ*M (Jurkat) treatment with NaD1, or 4 h after exposure to UV radiation. Data represent two independent experiments. (**c**) The ability of the pancaspase inhibitor, Q-VD-OPH, to inhibit the cytotoxic activity of NaD1 towards MM170 and Jurkat cells was determined over 6, 16h and 24 h following 1.25 *μ*M (MM170) or 2.5 *μ*M (Jurkat) treatment with NaD1. For MM170 cells – 6 h, *P*=0.686; 16 h, *P*=0.375; 24 h, *P*=0.491. For Jurkat cells – 6 h, *P*=0.977; 16 h, *P*=0.092; 24 h, *P*=0.485, unpaired Student’s two-tailed *t*-test. (**d**) To test directly the ability of Q-VD-OPH to inhibit apoptosis, phosphatidylserine exposure as determined by FITC-Annexin V staining of Jurkat cells 4 h after exposure to UV radiation was detected via flow cytometry. *P*=0.011, unpaired Student’s two-tailed *t*-test. Data in (**a**), (**c**) and (**d**) are representative of at least two independent experiments, error bars represent S.E.M, *n*=3. *P<0.05.

To investigate the role of caspases in NaD1-mediated cytotoxicity, caspase activity was measured using a Caspase-Glo 3/7 Assay, which detects the activity of downstream effector caspases, caspase-3 and -7.^23^ NaD1 at subacute concentrations did not promote the activation of effector caspases in MM170 or Jurkat cells, across the three time points tested (Figure 4a). In contrast, cells treated with UV to induce apoptosis robustly induced caspase activation after 4 h (Figure 4a).

A known hallmark of late-stage apoptosis is the caspase-3-dependent cleavage of genomic DNA into ~180 bp fragments and multimers thereof, which can be detected via agarose gel electrophoresis and appear as ‘laddering’.^24,25^ To further examine the potential role of caspases in NaD1-mediated cytotoxicity and whether NaD1 induces DNA fragmentation in tumor cells, the DNA fragmentation assay was performed at 6, 16 and 24 h after NaD1 treatment. Cells were treated with concentrations of NaD1 similar to the IC_50_ value of each cell type over 24 h (1.25 *μ*M for MM170, 2.5 *μ*M for Jurkat). Whereas a distinct laddering effect, representing DNA fragmentation, was observed for the UV-treated samples of both cell types, no laddering was detected at any of the time points for either cell type following NaD1 treatment (Figure 4b).

Next, to determine whether NaD1-mediated cytotoxicity of tumor cells is affected by caspase inhibition, cells were pre-treated with the pancaspase inhibitor, Q-VD-OPH, before NaD1 treatment at subacute concentrations as described for Figure 4b. Q-VD-OPH did not have a significant effect on NaD1-mediated membrane permeabilization for either cell type (Figure 4c). As a positive control, Q-VD-OPH inhibited UV-induced apoptosis in Jurkat cells, as measured by flow cytometry (Figure 3d).

Taken together, these data indicate that caspases do not have a role in NaD1-mediated cell killing of the cell lines tested, at subacute concentrations.

#### NaD1 induces a necrotic cell phenotype in MM170 cells

**Figure 5:**
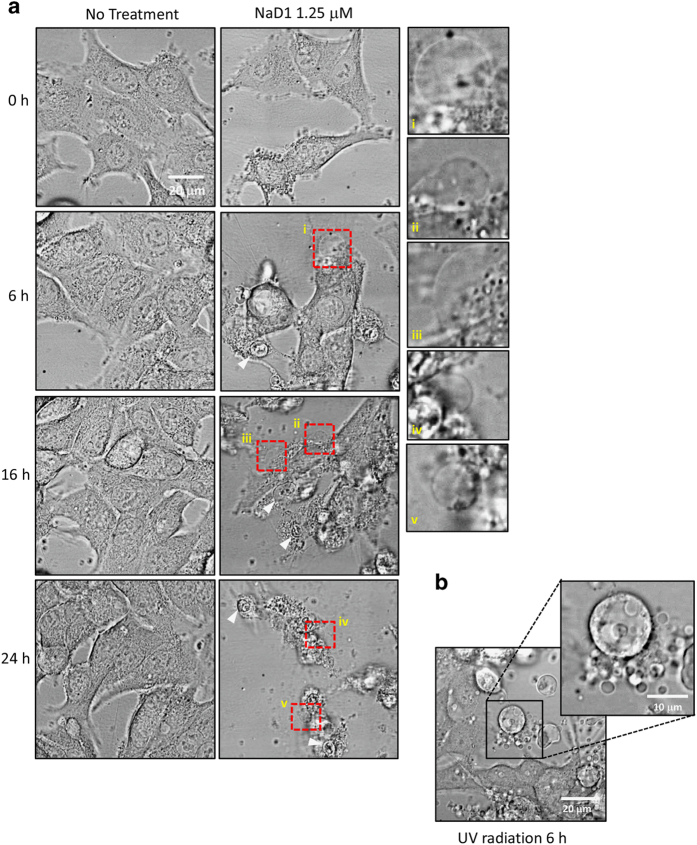
MM170 cells display a necrotic phenotype following subacute treatment of NaD1 over 24 h. Morphological changes in MM170 cells following treatment with NaD1 (1.25 *μ*M) or ultraviolet (UV) radiation were examined via DIC live cell imaging by spinning disk confocal microscopy. (**a**) NaD1-treated cells were imaged over 24 h, with images recorded at 0, 6, 16 and 24 h time points. Scale bars in (**a**) represent 20 *μ*m. (**b**) MM170 cells exposed to UV radiation were imaged 6 h after exposure. Data are representative of three independent experiments.

To monitor changes in tumor cell morphology following treatment with a subacute concentration of NaD1, live cell imaging of MM170 cells was performed. MM170 cells were treated with 1.25 *μ*M NaD1 over 24 h, with DIC images captured at 0, 6, 16 and 24 h time points (Figure 5a). MM170 cells treated with UV radiation to induce apoptosis were also imaged at 6 h as a control (Figure 5b). At all three time points over 24 h following NaD1 treatment, large plasma membrane blebs were observed in NaD1-treated cells (red boxes, enlarged panels), along with the appearance of nuclear swelling (white arrows), indicating necrosis (Figure 5a). In contrast, apoptotic cell morphologies including cell rounding and the appearance of multiple small membrane blebs were observed in MM170 cells following UV treatment (Figure 5b). These data indicate that, at time points over 24 h at which NaD1-mediated plasma membrane permeabilization can be observed, subacute treatment of NaD1 also induces necrotic morphologies in MM170 cells.

#### NaD1-mediated cell killing is inhibited by a PIP2-sequestering agent but not by a necroptosis inhibitor

**Figure 6:**
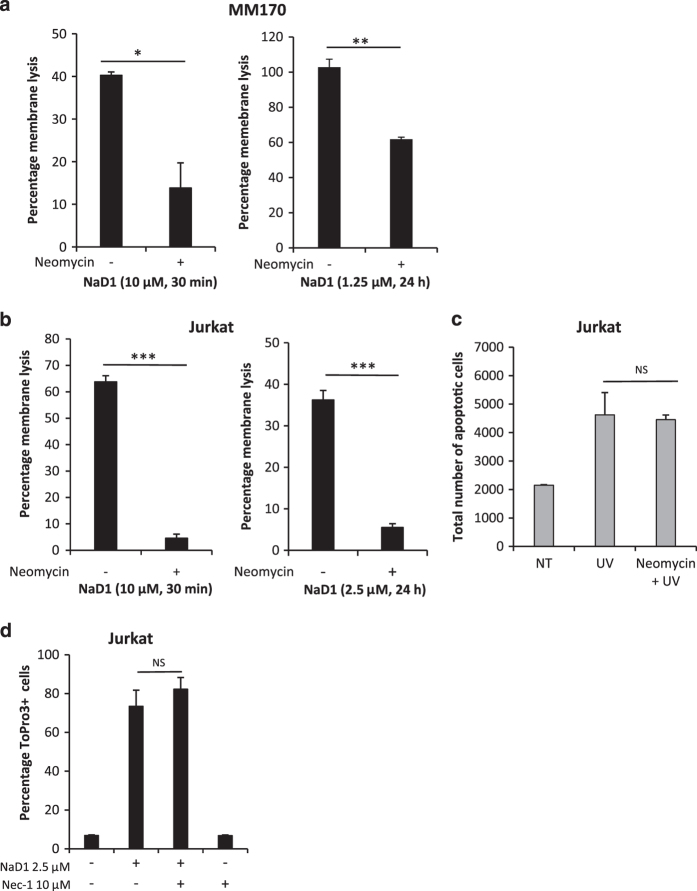
The cytotoxic activity of NaD1 at subacute concentrations over 24 h is significantly inhibited by neomycin but not necrostatin-1. The ability of the PIP2-sequestering agent, neomycin, to inhibit NaD1-mediated LDH release from MM170 and Jurkat cells was determined using the LDH cytotoxicity assay. MM170 (**a**) and Jurkat (**b**) cells were pre-treated with 10 mM neomycin for 1 h before the addition of NaD1 at 1.25 *μ*M (MM170) or 2.5 *μ*M (Jurkat) for 24 h. MM170, *P*=0.011, Jurkat, *P*=0.001, unpaired Student’s two-tailed *t*-test. Treatment with 10 *μ*M NaD1 over 30 min was also performed on both cell lines as a comparison. MM170, *P*=0.000, Jurkat, *P*=0.000, unpaired Student’s two-tailed *t*-test. (**c**) To test directly the ability of neomycin to inhibit apoptosis, phosphatidylserine exposure as determined by FITC-Annexin V staining of Jurkat cells 4 h after exposure to ultraviolet (UV) radiation was detected via flow cytometry. *P*=0.910, unpaired Student’s two-tailed *t*-test. Data are representative of two independent experiments, error bars represent S.E.M, *n*=3. (**d**) The ability of necrostatin-1 (Nec-1) to inhibit NaD1-mediated membrane permeabilization was investigated. Cells pre-treated with 10 *μ*M necrostatin-1 (Nec-1) were subjected to 24 h treatment with 2.5 *μ*M NaD1 and analyzed by flow cytometry, with percentage of cell death indicated by ToPro3-positive staining. *P*=0.437, unpaired Student’s two-tailed *t*-test. Data are representative of three independent experiments, error bars represent S.E.M, *n*=3. *P<0.05, **P<0.01, ***P<0.001.

NaD1 at an acute concentration (10 *μ*M) has been previously shown to target PIP2 at the plasma membrane of tumor cells to elicit membrane permeabilization, a process that can be inhibited by the PIP2-sequestering agent, neomycin.^26,27^ To determine whether PIP2 is also involved in the induction of NaD1-mediated cell killing at subacute concentrations over time, LDH cytotoxicity assays were performed on MM170 and Jurkat cells following 24 h of treatment with NaD1 at subacute concentrations in the presence or absence of neomycin. As a comparison, the effects of neomycin on cells treated with 10 *μ*M NaD1 over 30 min, was also included. Pre-treatment with 10 mM neomycin significantly inhibited NaD1-mediated cytotoxicity in MM170 (Figure 6a) and Jurkat (Figure 6b) cells at both acute and subacute concentrations over 30 min or 24 h time points. In contrast, neomycin did not inhibit UV-induced apoptosis in Jurkat cells, as measured by flow cytometry (Figure 6c). These data indicate that NaD1-mediated induction of cell death in tumor cells at subacute concentrations occurs via a PIP2-dependent mechanism.

Last, to determine if the the mechanism of NaD1-mediated membrane permeabilization involves necroptosis, Jurkat cells were treated with the RIP1 kinase inhibitor, necrostatin-1, before 24 h NaD1 treatment and analysis by flow cytometry (Figure 6d). Necrostatin-1 failed to inhibit NaD1-mediated membrane permeabilization of Jurkat cells under subacute conditions, thereby suggesting that NaD1 is unlikely to activate a necroptotic pathway.

Collectively, these data indicate that NaD1 does not activate an apoptotic or necroptotic cell death pathway in either MM170 or Jurkat cells over 24 h, at the subacute concentrations tested.

## Discussion

The anticancer activity of several CAPs, including defensins, has been shown to depend on the ability of a peptide to interact with the tumor cell plasma membrane, inducing membrane disruption or permeabilization.^17,21,26,28–30^ In addition to the induction of physical membrane damage leading to primary necrosis, some CAPs have also demonstrated the ability to internalize and/or interact with intracellular targets, thereby activating other cell death pathways such as apoptosis.^14,21^ While the ability of NaD1 to induce rapid, necrotic cell death in mammalian tumor cells has been characterized previously,^17^ the effects of NaD1 on mammalian tumor cells over a broader range of concentrations (for example, subacute concentrations) and treatment times have not been investigated. Herein, we show that treatment of human tumor cells with subacute concentrations of NaD1 resulted in a marked loss of cell viability but without the rapid (<30 min) membrane permeabilization that is characteristic of acute levels of NaD1. To determine if this loss in cell viability was due to apoptosis, subacute treatment of adherent and non-adherent tumor cell lines with NaD1 was investigated, revealing that, under these conditions, NaD1 does not induce apoptosis over a 24 h period. Furthermore, the inhibition of NaD1 activity by neomycin indicates that the previously reported PIP2-mediated mechanism of membrane destabilization may also apply within these time and concentration parameters.

The membrane-permeabilizing activity of NaD1 in mammalian tumor as well as fungal cells has been demonstrated to involve interaction with the plasma membrane phosphatidylinositol phospholipid, PIP2, with which NaD1 can form an oligomeric complex to mediate cell lysis.^17^ Biophysical and cell biology studies involving NaD1 and PIP2 have indicated that this lipid-mediated interaction is capable of destabilizing the plasma membrane of tumor cells, leading to acute cell injury and resulting in primary necrosis.^17^ It should be noted that although, in this study, the ability of NaD1 to inhibit fungal growth was abrogated by mutating key PIP2-binding residues, a more recent investigation suggests that PIP2 targeting by NaD1 is likely to be only one of several factors that contribute to fungal cell killing by NaD1.^31^ In the present study, the ability of NaD1 to inhibit markedly growth of mammalian tumor cells over 24 h at low concentrations (at which only low-level membranolytic activity was observed over 30 min) was demonstrated, presenting the possibility that the observed decrease in cell viability at subacute concentrations of NaD1 could be associated with the induction of apoptosis. This was further probed via investigating the role of caspases as well as changes to intracellular and membrane morphology following subacute treatment with NaD1.

The intrinsic pathway of apoptosis involves mitochondrial membrane permeabilization, either triggered by intracellular stress signals or otherwise, that can result in the release of cytochrome *C* from the mitochondria and activation of the apoptosome.^32^ Mitochondrial targeting has been implicated in the mechanisms of tumor cell cytotoxicity by a number of CAPs.^12,14,20,21^ For example, the *β*-hairpin peptide, bovine lactoferricin B, internalizes and induces mitochondrial membrane damage before induction of DNA fragmentation in Jurkat cells,^14^ while the bovine cathelicidin, BMAP-28, induces a loss of mitochondrial membrane potential in U937 cells that precedes plasma membrane permeabilization.^20^ Although we have previously observed that NaD1 binds to the mitochondrial membrane lipid, cardiolipin, *in vitro* and binds to intracellular organelles of permeabilized cells,^17^ our findings within the present study, which showed a simultaneous loss of MMP and cytosolic uptake of 4 kDa FITC-dextran, do not support the notion that NaD1-mediated cell killing is a mitochondria-dependent process. It is feasible that the observed loss in MMP could be, instead, due to a secondary effect of plasma membrane rupture and resultant loss in osmotic homeostasis, leading to organelle damage.

Caspase activation, which is a major hallmark of apoptosis, has been implicated in the anticancer mechanism of several other CAPs, including those that also display membranolytic properties. For example, the *α*-helical cecropia moth peptide, cecropin, induces elevated expression of caspase-3 and -8, as well as Fas and Fas-L in BEL-7402 hepatocellular carcinoma cells, indicating extrinsic apoptosis pathway activation,^33^ whereas the avocado defensin, PaDef, which is structurally related to NaD1^34^ induces the expression of caspase-7 and -9, as well as intrinsic pathway markers cytochrome *C* and Apaf-1.^12^ In TSU prostate cancer and B16 mouse melanoma cells, the *β*-hairpin horseshoe crab-integrin peptide conjugate, tachyplesin-RGD activates caspase-8 and -9, suggesting that this peptide has an effect on both intrinsic and extrinsic pathways,^35^ with similar observations made in U937 cells treated with the *α*-helical grouper fish peptide, epinecidin-1.^13^ In addition to evidence of caspase activation, inhibition of cell death has also been achieved in the presence of pancaspase inhibitors. For example, in MCF-7 cells treated with MG2B, a peptide conjugate from the *α*-helical African claw frog peptide, Magainin, and bombesin and in HeLa cells treated with the magainin-penetratin conjugate (MG2A), cell death was significantly reduced in the presence of the pancaspase inhibitor Z-VAD-fmk.^21,36^ In contrast, in this study a role for caspases in the cytotoxic mechanism of NaD1 under acute settings with MM170 and Jurkat cells was not observed. This was shown by the inability of (i) NaD1 to induce effector caspase activity and (ii) the pancaspase inhibitor Q-VD-OPH to inhibit NaD1-mediated membrane permeabilization, over 6–24 h.

A number of those CAPs that can induce apoptosis in mammalian tumor cells have also been shown to trigger the caspase-3-dependent process of DNA fragmentation.^25^ These include buforin IIb,^37^ BMAP-28,^20^ epinecidin-1^13^ and lactoferricin B.^14,38^ Consistent with the lack of caspase activity observed during this study, NaD1 failed to induce DNA laddering at any of the tested time points at subacute concentrations, further indicating that NaD1 does not mediate apoptosis in MM170 or Jurkat cells, under the conditions tested.

An additional key feature of late-stage apoptosis is the process of apoptotic cell disassembly, during which apoptotic cells undergo blebbing to form multiple, small, dynamic blebs.^39,40^ This phenotype is distinct from the unregulated event of necrotic cell death, which is marked by membrane permeability accompanied by organellar swelling^41–43^ and large, static (non-retracting) membrane blebs resulting from ionic leakage or influx.^40^ Under acute treatment conditions, necrotic blebbing of this nature has been previously observed in a number of adherent and non-adherent NaD1-treated tumor cell types, as well as in cells treated with the closely related Tomato defensin, TPP3 under the same conditions.^17,26^ In our microscopy studies, similar morphologies to these were observed in MM170 cells treated with subacute concentrations of NaD1. These were markedly distinct from cells treated with UV radiation, which adopted classic apoptotic blebbing, as described above. Based on these observations, it can further be inferred that subacute treatment with NaD1 does not mediate apoptosis.

Taken together, these data strongly suggest that the mechanism by which mammalian tumor cells undergo NaD1-mediated cell death at subacute concentrations is non-apoptotic and likely to be via primary necrosis as observed previously, under acute killing conditions.^17^ It should be noted that alternative non-apoptotic cell death pathways of pyroptosis and necroptosis have also been considered as potential pathways of NaD1-mediated tumor cell killing. However, as pyroptosis is a caspase-1-dependent process^44^ and Q-VD-OPH, which can inhibit several caspases including caspase-1, was unable to inhibit NaD1-mediated cytotoxicity, the role of pyroptosis in NaD1-mediated cell death pathway can be excluded. Similarly, necrostatin-1, which inhibits necroptosis via upstream blocking of RIP1 kinase,^45^ failed to inhibit NaD1-mediated membrane permeabilization in NaD1-treated Jurkat cells, suggesting that necroptosis does not have a role in NaD1-mediated cytotoxicity. However, it should be noted that, although inhibition of RIP1 kinase by necrostatin-1 did not have an effect on the cytotoxic activity of NaD1, the ability of NaD1 to effect the necroptotic pathway downstream of RIP1 kinase activity, such as via direct modulation of MLKL-mediated membrane disruption,^46^ not addressed in our current study should be determined to eliminate definitively necroptosis as having a role. Furthermore, as the PIP2-binding agent, Neomycin, was able to inhibit significantly NaD1-mediated membrane permeabilization even at these lower concentrations over a 24 h period, it is likely that PIP2-mediated oligomerization and membrane disruption of tumor cells, as described previously by Poon *et al*.,^17^ can occur under these conditions. The specific mechanism by which NaD1 monomers penetrate tumor cell membranes to gain access to inner-leaflet PIP2 remains to be determined. However, it is reasonable to consider that the degree of internalization and subsequent accumulation on the inner membrane surface of tumor cells by NaD1 is dependent on concentration, with subacute levels simply taking a longer time to reach a threshold level to enable oligomerization sufficient to destabilize the membrane. Further investigations are required to confirm the involvement of PIP2 in NaD1-mediated tumor cell killing at subacute concentrations and to determine whether other related defensins have similar effects on mammalian tumor cells.

## Materials and Methods

### Cell lines

Human epithelial cervical cancer (HeLa), leukemic T-cell (Jurkat), malignant melanoma (MM170) and monocytic lymphoma (U937) cell lines were cultured in RPMI-1640 medium (Invitrogen, Carlsbad, CA, USA). All culture media were supplemented with 5–10% fetal calf serum, 100 U/ml of penicillin and 100 *μ*g/ml of streptomycin (Invitrogen). Cell lines were cultured at 37 °C in a humidified atmosphere containing 5% CO_2_. Adherent cell lines were detached from the flask by adding a mixture containing 0.25% trypsin and 0.5 *μ*M EDTA (Invitrogen).

### Expression and purification of NaD1

The mature defensin domain of NaD1 was expressed as secreted recombinant protein in the methylotropic yeast *Pichia pastoris* and purified using an SP Sepharose column (GE Healthcare, Buckinghamshire, UK) as described previously.^47^

### MTT/MTS growth inhibition assay

Mammalian cells were seeded in triplicate into wells of a flat-bottomed 96-well microtitre plate (50 *μ*l) and incubated overnight at 37 °C under a humidified atmosphere containing 5% CO_2_/95% air. Complete culture medium (100 *μ*l) was then added to each well containing defensin and further incubated at 37 °C for 24 h. Background control wells containing the same volume of complete culture medium were included in the assay. For adherent cells, the cell viability MTT (Sigma-Aldrich, St Louis, MO, USA) assay was performed as follows: the MTT solution (1 mg/ml) was added to each well (100 μl) and the plate incubated for 2–3 h at 37 °C under a humidified atmosphere containing 5% CO_2_/95% air. Subsequently, the media were removed and replaced with dimethyl sulfoxide (DMSO; 100 μl; Sigma-Aldrich), and placed on a shaker for 5 min to dissolve the tetrazolium salts. For non-adherent cells, the cell viability MTS (Sigma-Aldrich) assay was performed as follows: 20 *μ*l of MTS solution (diluted 1 : 20 with the electron coupling reagent, phenazine methosulfate, according to the manufacturer’s instructions) was added to each well and incubated for 4 h. Absorbance of each well was then measured at 490 nm. Absorbance (MTT=570 nm; MTS=490 nm) was measured using a SpectraMax M5e Plate Reader (Molecular Devices, Sunnyvale, CA, USA) and analyzed using the SoftMaxPro 5.2 software (Molecular Devices), with IC_50_ values (the protein concentration to inhibit 50% of cell growth) determined by extrapolation using Excel.

### LDH cytotoxicity assay

LDH Cytotoxicity Assay Kit II (Abcam, Cambridge, UK) was used according to the manufacturer’s instructions to detect the release of the cytosolic enzyme, LDH, from MM170 and Jurkat cells following treatment with NaD1, described previously.^17^ MM170 cells were seeded 24 h before experimentation and Jurkat cells were seeded on the day of experimentation. Assays for both cell types were performed in 96-well plate format and cells were incubated in complete culture medium at 37 °C under a humidified atmosphere containing 5% CO_2_/95% air during treatments with NaD1. Cells were then centrifuged at 600×*g* and the supernatant was added to LDH reaction mix for 30 min at RT. The absorbance of the enzymatic product at 450 nm was measured using a SpectraMax M5e Plate Reader and analyzed using the SoftMaxPro 5.2 software. In some experiments, cells were pre-treated with 10 mM neomycin (Sigma-Aldrich) for 30 min, or 50 *μ*M Q-VD-OPH (SM Biochemicals, Aneheim, CA, USA) or DMSO (equivalent volume) for 1 h, before the addition of NaD1.

### Caspase-Glo 3/7 assay

Detection of caspase activity following treatment of NaD1 was determined using the Caspase-Glo 3/7 Assay (Promega, Madison, WI, USA), according to the manufacturer’s instructions. MM170 and Jurkat cells were seeded in complete culture medium (50 *μ*l) in an opaque 96-well plate, with MM170 cells seeded 24 h before experimentation and Jurkat cells seeded on the day of experimentation. Cells were then treated with either NaD1 or exposed to UV radiation using a Stratalinker UV crosslinker (Stratagene, La Jolla, CA, USA) and incubated at 37 °C under a humidified atmosphere containing 5% CO_2_/95% air. Cells were then allowed to equilibrate to room temperature for 10 min before the addition of Caspase-Glo 3/7 reagent (50 *μ*l). Cells were then incubated at RT in dark for 1–3 h. Caspase activity, as determined by luminescence, was then measured using a SpectraMax M5e Plate Reader, with the resultant data analyzed using the SoftMaxPro 5.2 software (Molecular Devices).

### FITC-Annexin V and ToPro3 staining via flow cytometry

For experiments involving the ability of necrostatin-1 to inhibit NaD1-mediated cell killing, Jurkat cells (1×10^5^ cells per sample in complete culture medium) were pre-treated with 10 *μ*M necrostatin-1 for 30 min before 24 h treatment with 2.5 *μ*M NaD1. Cells were then incubated for 10 min with 1 *μ*M TO-PRO-3 nucleic acid stain and analyzed on a FACS Canto II (BD Biosciences, San Jose, CA, USA) with at least 10 000 events captured for each sample, followed by analysis with the FlowJo software (Tree Star Inc, Ashland, OR, USA). Dead cell populations were determined by gating TO-PRO-3 positively stained cells. For control experiments in which the ability of neomycin or Q-VD-OPH to inhibit apoptosis was confirmed, Jurkat cells (8×10^4^ cells per sample in complete culture medium) were pre-treated with either neomycin (10 mM, 30 min prior) or Q-VD-OPH (50 *μ*M, 1 h prior) before exposure to UV radiation for 4 h. Cells were then incubated for 10 min in the dark with 100 *μ*l 2× Annexin V binding buffer containing FITC-Annexin V stain (diluted 1/100) and 1 *μ*M TO-PRO-3 nucleic acid stain. Samples were then analyzed on a FACS Canto II with apoptotic cell populations by gating TO-PRO-3 intermediate/low, FITC-Annexin V-positive cells, according to a method described previously.^39^

### DNA fragmentation assay

To determine whether NaD1 is able to induce DNA fragmentation in mammalian tumor cells, a DNA fragmentation assay was performed over 6, 16 and 24 h time points on MM170 and Jurkat cells. A total of 5×10^5^ cells per condition were seeded into 60 mm sterile round tissue culture dishes, with MM170 cells being seeded 24 h before experimentation and Jurkat cells seeded on the day of experimentation. Cells were then treated with NaD1 for desired times or exposed to UV radiation (and analyzed 4 h after exposure) before being transferred from dishes to microfuge tubes and pelleted by centrifugation at 2000 r.p.m. for 5 min at 4 °C. Supernatant was then removed (MM170 cells were lifted with 0.25% trypsin and 0.5 μM EDTA before centrifugation and washed once additionally with 1x PBS). Twenty microliters of TES lysis buffer was then added to each of the cell pellets followed by 10 *μ*l RNase A/T1 Mix (Thermo Fisher, Scoresby, VIC, Australia), flicking the base of tubes to mix. Samples were incubated at 37 °C for 1 h. Ten microliters of proteinase K, RNA grade (Invitrogen) was then added to each sample and incubated at 50 °C for 90 min. Five microliters of 6× DNA loading dye was then added to each sample. Samples were loaded into wells of a 1.5% agarose gel in TBE containing SYBR Safe (Thermo Fisher) and run at 35 V for 4 h. DNA laddering on agarose gels was then detected on a G:BOX (Syngene, Cambridge, UK) under UV and analyzed using the Genesys software (Genesys, Brisbane, QLD, Australia).

### Live cell imaging

For all microscopy experiments, MM170 cells were cultured on 4-well Nunc Lab-Tek (Nunc, Germany) chamber slides and allowed to adhere for 24 h before imaging. DIC live cell imaging was performed on a Zeiss spinning disk confocal microscope (Zeiss, Oberkochen, Germany) using a 63× oil immersion objective in a 37 °C/5% CO_2_ atmosphere. Cells were imaged in growth media in the presence of 1.25 *μ*M NaD1 at specified time points over 24 h. Image processing and data analysis were performed using the ZEN imaging software (Zeiss). Fluorescence live cell imaging was performed on a Zeiss LSM-780 Confocal Laser Scanning Microscope (Zeiss) using a ×63 oil immersion objective in a 37 °C/5% CO_2_ atmosphere. Cells were preincubated with mitochondrial membrane potential-dependent mitochondrial stain, MTR (Thermo Fisher) at 50 nM in the growth medium for 15 min at 37 °C before imaging. Cells were then imaged in media containing 4 kDa FITC-dextran at 10 *μ*g/ml, in the presence of 1.25 *μ*M NaD1 over 4 h at 30 s intervals. Image processing and data analysis were performed using the ZEN software (Zeiss). Kinetic analysis of FITC-dextran uptake *versus* loss in MTR fluorescence was performed in Excel with mean fluorescence intensities in individual cells captured across multiple imaging sessions and displayed as average across all cells.
