# Supplementary material for: The plant defensin NaD1 induces tumor cell death via a non-apoptotic, membranolytic process
Source: Cell Death Discov. 2017 Jan 23;3:16102–. doi: 10.1038/cddiscovery.2016.102 (PMC5253418; doi:10.1038/cddiscovery.2016.102)
Supplement: Supplementary Information [file cddiscovery2016102-s1.doc]

# The plant defensin NaD1 induces tumor cell death via a non-apoptotic, membranolytic process

AA Baxter, IKH Poon* & MD Hulett*

Department of Biochemistry and Genetics, La Trobe Institute for Molecular Science, La Trobe University, Melbourne, Victoria 3086, Australia. * These authors contributed equally to this work. Correspondence should be addressed to IKPH (i.poon@latrobe.edu.au) or MDH ([m.hulett@latrobe.edu.au](mailto:m.hulett@latrobe.edu.au)).

# Supplementary Information

**Video S1. Live cell imaging of NaD1-mediated mitochondrial membrane permeabilization and FITC-Dextran uptake in MM170 cells.** Live cell imaging time course was performed on MM170 cells and captures point of membrane permeabilization in two cells following NaD1 treatment. Cells were pre-treated with MTR before being treated with 1.25 μM NaD1 and imaged at 30 s intervals over 4 h in growth medium containing FITC-dextran (4 kDa). Relative time (top left) indicates time from addition of NaD1. Data is representative of three independent experiments.
